# Taxifolin Activates the Nrf2 Anti-Oxidative Stress Pathway in Mouse Skin Epidermal JB6 P+ Cells through Epigenetic Modifications

**DOI:** 10.3390/ijms18071546

**Published:** 2017-07-17

**Authors:** Haixue Kuang, Zhenqiu Tang, Chengyue Zhang, Zhibin Wang, Wenji Li, Chunjuan Yang, Qiuhong Wang, Bingyou Yang, Ah-Ng Kong

**Affiliations:** 1 Key Laboratory of Chinese Materia Medica, Heilongjiang University of Chinese Medicine, Harbin 150040, China; hxkuang@hljucm.net (H.K.); dzl8161921@gmail.com (Z.T.); qhwang@hljucm.net (Q.W.); ybywater@hljucm.net (B.Y.); 2 Department of Pharmaceutics, Ernest Mario School of Pharmacy, Rutgers, The State University of New Jersey, Piscataway, NJ 08854, USA; chengyue.zhang@gmail.com (C.Z.); wl365@scarletmail.rutgers.edu (W.L.); chunjuanyang@hrbmu.edu.cn (C.Y.); 3 College of Pharmacy, Harbin Medical University, Harbin 150086, China

**Keywords:** skin cancer, Nrf2, epigenetics, taxifolin, JB6 P+ cells

## Abstract

Nuclear factor erythroid-2 related factor 2 (Nrf2) is a vital transcription factor that regulates the anti-oxidative defense system. Previous reports suggested that the expression of the *Nrf2* gene can be regulated by epigenetic modifications. The potential epigenetic effect of taxifolin (TAX), a potent cancer chemopreventive agent, in skin cancer chemoprotection is unknown. In this study, we investigated how Nrf2 is epigenetically regulated by TAX in JB6 P+ cells. TAX was found to inhibit the 12-*O*-tetradecanoylphorbol-13-acetate (TPA)-induced colony formation of JB6 P+ cells. TAX induced antioxidant response element (ARE)-luciferase activity in HepG2-C8 cells and up-regulated mRNA and protein levels of Nrf2 and its downstream genes heme oxygenase-1 (HO-1) and NAD(P)H quinone oxidoreductase 1 (NQO1), in JB6 P+ cells. Furthermore, bisulfite genomic sequencing revealed that TAX treatment reduces the methylation level of the first 15 CpGs sites in the Nrf2 promoter. Western blotting showed that TAX inhibits the expression levels of DNA methyltransferase (DNMT) and histone deacetylase (HDAC) proteins. In summary, our results revealed that TAX can induce expression of Nrf2 and its downstream target genes in JB6 P+ cells by CpG demethylation. These finding suggest that TAX may exhibit a skin cancer preventive effect by activating Nrf2 via an epigenetic pathway.

## 1. Introduction

Reactive oxygen species (ROS) are highly proactive molecules originating from free radicals and molecular oxygen [1]. Oxidative stress is considered as the imbalance between pro-oxidants and anti-oxidants. Superfluous ROS production and impairment of cellular anti-oxidant defense system may cause endogenous oxidative stress [2]. ROS-induced oxidative stress has been implicated in the pathogenesis of carcinogenesis [3]. Skin cells are constantly exposed to ultra-violet (UV) radiation, carcinogens or mutagens, which could induce ROS generation in the skin. ROS overproduction within tissues or cells can damage DNA and lead to tumor initiation. UV radiation is regarded as a major environmental factor in the development of skin carcinogenesis [4]. The morbidity of skin cancer, such as squamous cell carcinoma and basal cell cancer, is conspicuously increasing worldwide [5,6]. In the USA, skin cancer is highly common, and approximately one million new cases are diagnosed each year [7]. Research on skin cancer and development of new prophylactic agents require urgent attention.

Nuclear factor erythroid-2 related factor 2 (Nrf2) is a vital transcription factor that promotes transcription of cytoprotective genes in response to oxidative stress, which is regulated by the adaptor protein Kelch-like ECH-associating protein 1 (Keap1) [8,9]. Under stress conditions, the interaction of Keap1 and Nrf2 is disrupted, and Nrf2 accumulates in the cell nucleus. Then, Nrf2 binds to the antioxidant response element (ARE) in the promoter region of some phase II enzyme genes and to expressed Nrf2 downstream enzymes [10,11]. These enzymes including heme oxygenase-1 (HO-1) and NAD(P)H quinone oxidoreductase 1 (NQO-1) that protect against oxidation. A large number of natural dietary compounds are thought to protect against oxidative stress. Some of these compounds have been shown to induce genes involved in cancer prevention by activating the Nrf2 pathway [8]. The critical role of Nrf2 induction via sulforaphane pretreatment involves the blocking of 12-*O*-tetradecanoylphorbol-13-acetate (TPA)-induced neoplastic transformation in JB6 P+ cells [12]. Besides, animal experiments have shown that Nrf2 knockout mice significantly increased susceptibility to various diseases, such as cancer [13,14].

“Epigenetics” involves the covalent modification of DNA, RNA, or protein, resulting in changes in the function or structure of these molecules, without changing their primary sequences [15]. The malignant transformation of normal cells is always actuated by genetic or epigenetic changes. With the development of the next-generation sequencing technique, it has become possible to profile the epigenomes and genomes of many primary tumors from almost all cancer types [16]. Epigenetic change influences nearly every process in tumorigenesis [17]. In a previous study, expression of Nrf2 was found to be regulated by epigenetic alterations in the prostate tissue of the transgenic adenocarcinoma mouse. It demonstrated that Nrf2 expression can also be adjusted by epigenetic changes in the adenocarcinoma mouse [18].

Many naturally occurring plant compounds have been evaluated as “chemopreventive agents” that can reduce the side effects of other anticancer therapeutic drugs, such as dietary flavonoids [19,20]. For instance, quercetin-3-methyl-ether, a natural product present in different kinds of plants, possesses potent anticancer activity [21]. Taxifolin (TAX), also named as dihydroquercetin, is a common flavonoid usually found in pinaceae plants, such as *Pseudotsuga taxifolia*, *Taxus chinensis*, *Cedrus deodara* and *Pinus roxburghii* [22,23]. Previous research also reported that citrus fruit and onions contained quite large amounts of TAX [24]. TAX has long been used clinically for treatment of cardiovascular and cerebrovascular diseases [25]. In recent years, TAX has been discovered to exert various pharmacological action, including anticancer, antioxidant, anti-inflammatory, and antibacterial activities [26,27,28]. TAX is a potent chemopreventive agent, which may be attributed to its ability to modulate antioxidant response pathway proteins and inflammation in tumor micro-environment [29]. In a previous report, TAX stimulated the expression of phase II antioxidant and detoxifying enzymes via the Nrf2-dependent pathway, and exerted a vital protective activity against DNA oxidative damage [30]. Importantly, TAX significantly enhances HO-1 expression by inducing Nrf2 expression in cytoplasm and nuclear translocation [31]. In addition, TAX can also markedly inhibit tumor morbidity by topical treatment of dorsal skin [32]. TAX reportedly exerts multiple biological effects including preventive effects in skin cancer. However, the direct target and molecular mechanisms of it in skin carcinogenesis chemoprevention are still unknown. Therefore, an in vitro study was performed to investigate the potential inhibitory effect of TAX on the neoplastic transformation of JB6 P+ cells, and to determine the underlying epigenetic mechanisms.

## 2. Results

### 2.1. Cytotoxicity of TAX in JB6 P+ Cells and HepG2-C8 Cells

**Figure 1:**
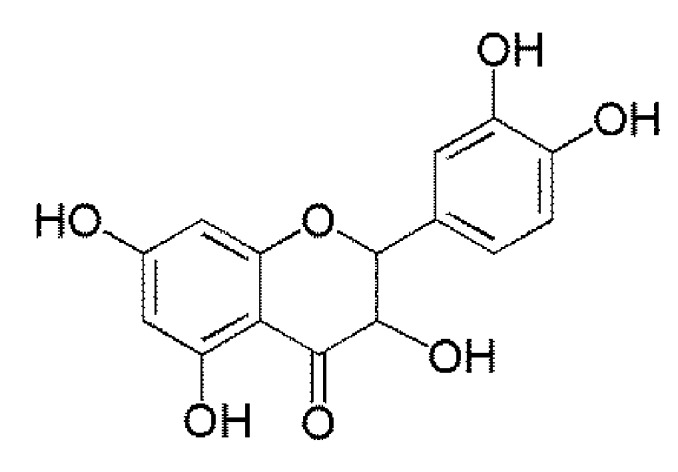
Chemical structure of taxifolin (TAX).

**Figure 2:**
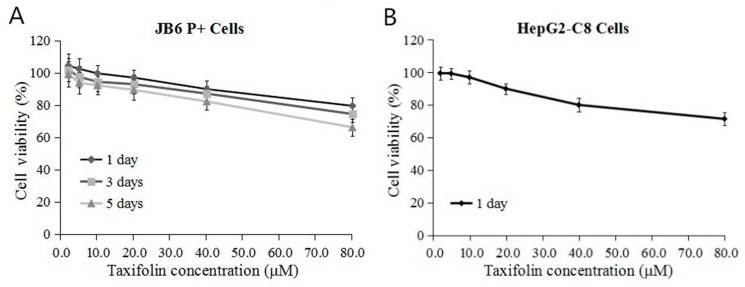
Cell viability of JB6 P+ cells and HepG2-C8 cells after treatment by TAX. After incubation for 24 h, the cells were treated with either dimethyl sulfoxide (DMSO) or different concentrations of TAX. Cell viability was determined using the 3-(4,5-dimethylthiazol-2-yl)-5-(3-carboxymethoxyphenyl)-2-(4-sulfophenyl)-2H-tetrazolium (MTS) assay with different subsequent treatments. (**A**) JB6 P+ cells were treated by TAX for one, three and five days at different concentrations (2.5 to 80 μM); (**B**) HepG2-C8 cells were treated by TAX for one day at different concentrations (2.5 to 80 μM). The data are expressed as the mean ± standard deviation (SD) (*n* = 3).

As the first step of our study, the cell viability of JB6 P+ cells and HepG2-C8 cells was analyzed to determine the cytotoxic effect of TAX using a [3-(4,5-dimethylthiazol-2-yl)-5-(3-carboxymethoxyphenyl)-2-(4-sulfophenyl)-2H-tetrazolium (MTS) assay. The chemical structure of TAX is shown in Figure 1. The results showed that TAX treatments decreased cell viability in JB6 P+ cells and HepG2-C8 cells in a dose dependent manner (Figure 2A,B). A low dose of TAX (<2.5 μM) was less toxic than the high-dose preparation (80 μM) in JB6 P+ cells. The viability of the cells treated with 40 μM TAX was greater than 80% in JB6 P+ cells and HepG2-C8 cells. Thus, TAX concentrations of 10 to 40 μM were utilized for further experiments in this study.

### 2.2. TAX Inhibits TPA-Induced JB6 P+ Cells and JB6-shNrf2 Cells Transformation

**Figure 3:**
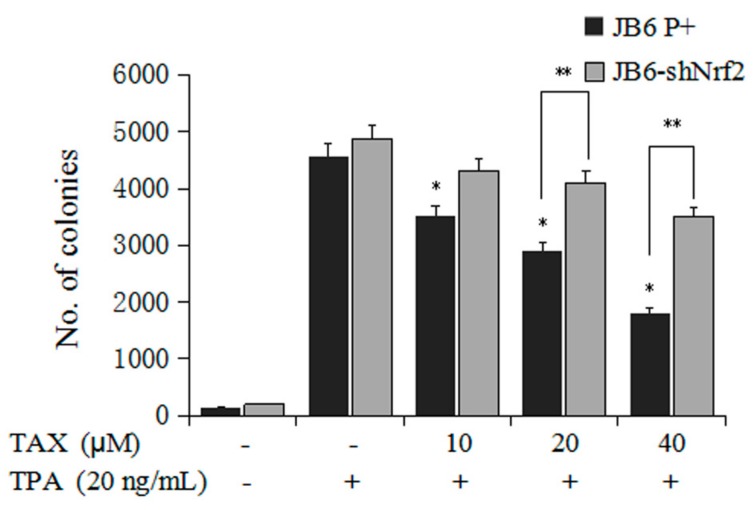
Inhibitory effect of TAX on the 12-*O*-tetradecanoylphorbol-13-acetate (TPA)-induced transformation of JB6 P+ and shNrf2-transfected JB6 P+ cells. The cells were maintained with TPA (20 ng/mL) alone or in a combination with TAX in a 5% CO_2_ incubator at 37 °C for 14 days. The colonies exhibiting anchorage-independent growth were counted under a microscope using ImageJ software. (National Institutes of Health, version 1.50a, http://rsbweb.nih.gov/ij). The graphical data are presented as the average of triplicate results from two independent experiments. * *p* < 0.05, indicating a significant decrease in colony formation relative to that of the JB6 P+ cells treated with TPA alone in soft agar; ** *p* < 0.05, indicating significant differences between the JB6 P+ cells group and JB6-shNrf2 cells group in the same treatment.

Second, we investigated inhibition of TAX to TPA-induced JB6 P+ cells and JB6-shNrf2 cells transformation. The effects of TAX treatment on the TPA-induced anchorage-independent growth of JB6 P+ cells and JB6-shNrf2 cells were evaluated in soft agar. TAX treatment with concentrations ranging from 10 to 40 μM observably decreased the number of JB6 P+ colonies relative to those of the TPA-treated control group (Figure 3). The result indicates that TAX may exert a potential preventive effect against TPA-induced carcinogenesis in JB6 P+ cells. On the other hand, the colony formation of JB6-shNrf2 cells in soft agar was significantly increased when compared with the JB6 P+ cell line in the same treatment (Figure 3), but no significant difference was observed between the TPA-treated control group and the TAX treatment group. The results indicated that the protective effect of TAX slowed down in the JB6-shNrf2 cells.

### 2.3. TAX Induces ARE-Luciferase Reporter Activity

**Figure 4:**
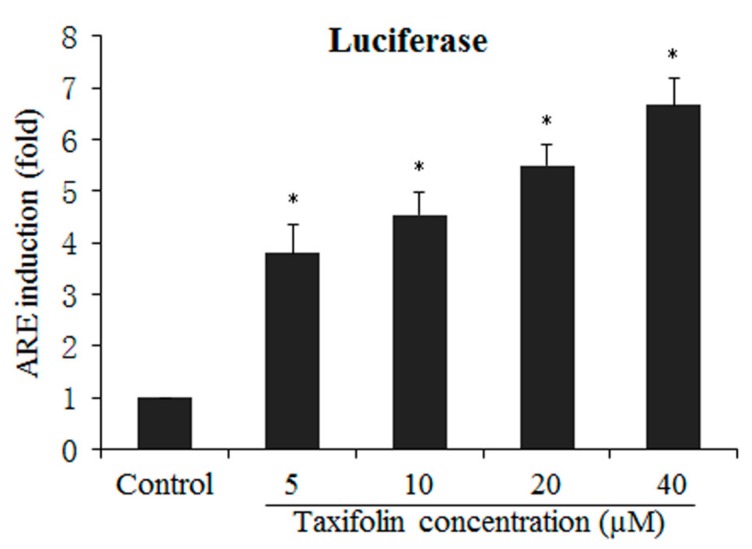
Induction of antioxidant response element (ARE)-luciferase activity by TAX treatment at concentrations from 5 to 40 μM in HepG2-C8 cells expressed with ARE-luciferase vector. The cells were treated with TAX 5 to 40 μM for 24 h. The normalization of the luciferase activity was performed on the basis of protein concentrations. The data were obtained from three independent experiments and expressed as the inducible fold change relative to that of the vehicle control. * *p* < 0.01, indicating significant differences between the treatment and control groups.

Next, we evaluated the effect of TAX on Nrf2-ARE activation using HepG2-ARE-C8 cells. The relative luciferase activity in the cells transfected with the ARE-luciferase reporter vector in the treatment groups compared with the control group is shown in Figure 4. TAX induced luciferase activity in a dose-dependent manner at the concentrations ranging from 5 to 40 μM, although no inductive effect was observed at concentrations lower than 5 μM. This result further verified the effect of TAX on Nrf2, as reported previously.

### 2.4. TAX Upregulates the mRNA and Protein Levels of Nrf2 Target Enzymes in JB6 P+ Cells

**Figure 5:**
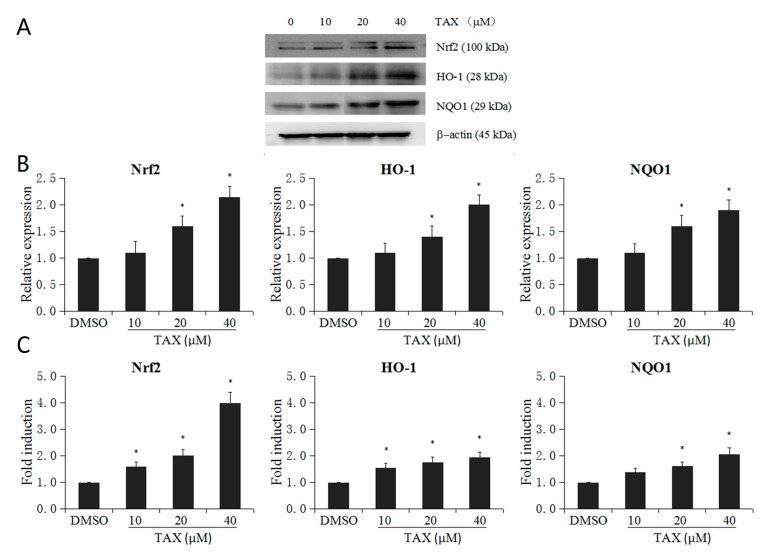
Effect of TAX on mRNA and protein expression of *Nrf2* and *Nrf2* target genes (HO-1 and NQO1) in JB6 P+ cells. JB6 P+ cells were treated with different TAX concentration (10 to 40 μM). (**A**) Western blot images of Nrf2 and its downstream genes including HO-1 and NQO1; (**B**) TAX increased the protein expression of Nrf2 and Nrf2 downstream enzymes; (**C**) TAX increased the mRNA levels of Nrf2 and its downstream representative enzymes including HO-1 and NQO1. The graphical data are presented as the mean ± SD from three independent experiments. * *p* < 0.05, indicating significant differences in each target protein or mRNA compared with its level in non-TAX-treated cells.

We also examined whether TAX could upregulate the protein and mRNA levels of Nrf2 target enzymes in JB6 P+ cells. Protein bands were densitometrically analyzed using ImageJ software (Figure 5A). Our results showed that TAX treatment significantly increased the mRNA expression of Nrf2, HO-1 and NQO1 (Figure 5C). The upregulation of Nrf2, HO-1 and NQO1 occurred in a dose-dependent manner at concentrations ranging from 10 to 40 μM. In accordance with the quantitative real-time PCR (qRT-PCR) results, TAX (10 to 40 μM) also increased the protein levels of Nrf2, HO-1 and NQO1 in a dose-dependent manner (Figure 5B). These experimental results indicate that TAX holds the potential to induce the Nrf2 pathway, including detoxifying and antioxidant enzymes.

### 2.5. TAX Decreases the Proportion of Methylated CpG in the Nrf2 Gene Promoter Region

**Figure 6:**
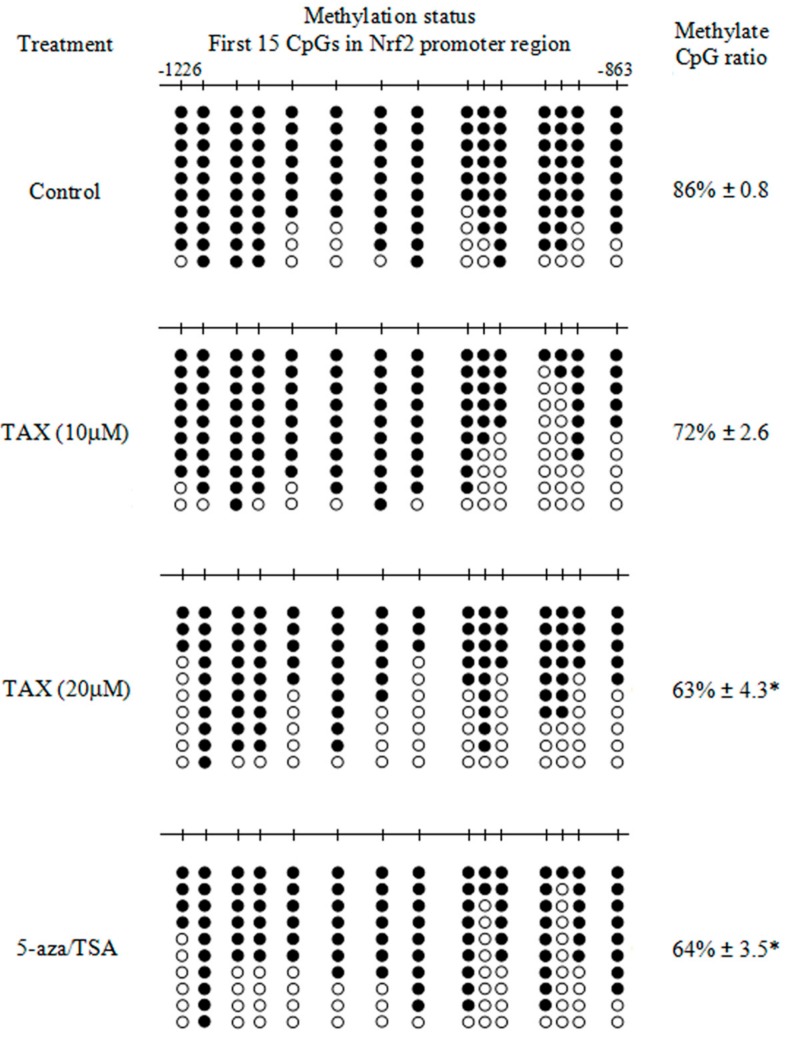
Methylation level at the 15 CpG sites (located between −1226 and −863) in the Nrf2 promoter determined by bisulfite genomic sequencing. The gene sequence of the first 15 CpG sites which located between −1226 and −863 was listed in the supplementary materials. The JB6 P+ cells were treated with different TAX concentration (10 to 20 μM) or 5-aza (250 nM) in minimum essential medium (MEM) containing 1% fetal bovine serum (FBS) for 5 days. Trichostatin A (TSA) (50 nM) was added to the medium on day 4. TAX (10 and 20 μM) and combination of 5-aza (250 nM)/TSA (50 nM) groups were compared with the DMSO control group. The comparison revealed significant differences (* *p* < 0.05) in methylation level. Black dots indicate methylated CpGs; open circles indicate the non-methylated CpGs. The 15 CpG sites correspond to the murine *Nrf2* gene with the translational start site defined as +1. The values of the methylated CpG ratio are the mean ± SD of at least 10 clones from three independent experiments.

To confirm whether TAX could decrease the proportion of methylated CpG in the *Nrf2* gene promoter region, the methylation status of the CpGs was determined by bisulfite sequencing. The Nrf2 promoter region containing the first 15 CpGs was converted and amplified. The hypermethylation of these 15 CpGs (methylation ratio, 86%) was observed in the control JB6 P+ cells (Figure 6), which was consistent with previous reports. In contrast to the control group, the methylation ratio decreased to 63% when cells were treated with TAX (20 μM). This result was similar to the positive control group (64%, 5-aza (500 nM) + TSA (100 nM)). These results suggest that TAX reverses the CpG methylation status in the *Nrf2* gene promoter, which may drive the transcriptional re-expression of Nrf2 in JB6 P+ cells.

### 2.6. TAX Inhibits the Protein Expression of Epigenetic Modification Enzymes in JB6 P+ Cells

**Figure 7:**
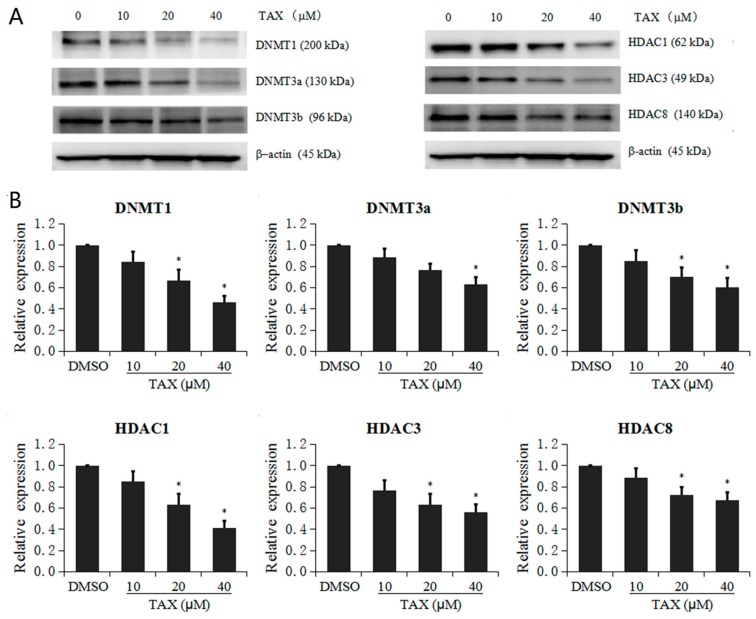
Effect of TAX (10 to 40 μM) on DNA methyltransferase (DNMT) and histone deacetylase (HDAC) protein expression in JB6 P+ cells. JB6 P+ cells were treated with different TAX concentrations (10 to 40 μM). The expression of DNMT1, 3a and 3b as well as HDAC1, 3, 4, and 8 proteins was detected by western blotting respectively. (**A**) Western blot images of DNMTs including DNMT1, 3a, and 3b, as well as HDAC1, 3, 4, and 8; (**B**) TAX significant inhibited the protein levels of DNMT1, 3a, and 3b, as well as HDAC1, 3, 4, and 8; The graphical data are represented as the mean ± SD from three independent experiments. * *p* < 0.05, indicating significant differences in each group compared with its level in non-TAX-treated cells.

Finally, the effects of TAX on DNA methyltransferase (DNMTs) (subtypes of DNMT1, 3a, and 3b) and histone deacetylases (HDACs) (subtypes of HDAC1-8) were further examined to investigate the epigenetic mechanism by which TAX affects promoter demethylation and induces *Nrf2* gene transcription. Protein bands of DNMTs and HDACs were densitometrically analyzed using ImageJ software (Figure 7A). The results confirm that TAX inhibits the protein expression of epigenetic modification enzymes in JB6 P+ cells. Different concentrations of TAX at 10 to 40 μM reduced the protein levels of DNMT1, 3a and 3b in a dose-dependent manner after treatment 5 days (Figure 7B). Consistent with the DNMTs expression, TAX also decreased the protein levels of HDAC1, 3 and 8 in a dose-dependent manner at concentrations ranging from 10 to 40 μM (Figure 7B). In addition, TAX repressed the expression of the HDAC2 and HDAC4 to HDAC7 protein, but no significant difference was observed. These results indicate that TAX holds the potential to epigenetically modify the DNA methylation of the Nrf2 promoter. This mechanism may be important for inducing Nrf2 as mentioned above.

## 3. Discussion

Varieties of flavonoids extracted from plants have been reported to have significant skin protective effects [33]. TAX, an active flavanonol, has been acknowledged as a strong chemopreventive agent for skin carcinogenesis, but the molecular mechanisms by which TAX shows preventive effects in skin cancer is still unknown. In our present study, we designed and performed a series of experiments to verify the antineoplastic potential of TAX and its pharmacological mechanism. We report that TAX directly inhibits TPA-induced cell transformation and increases Nrf2-ARE activity by upregulating HO-1 and NQO1 expression. Finally, we found that TAX decreases the methylated CpG ratio in the promoter region of *Nrf2* gene.

JB6 P+ is a normal skin keratinocyte cell line that transforms under carcinogenic or environmental challenges including TPA and UVB [34]. TPA exhibits a high affinity for protein kinase C (PKC) and induces activation of transcription factors, such as c-Fos, which regulates cell cycle and differentiation [35]. Therefore, the TPA-induced JB6 cell is usually used to establish neoplastic transformation model [36]. We used TPA as an inducer to stimulate tumor formation in JB6 P+ cells, and assessed the inhibitory effect of TAX on TPA-induced neoplastic transformation in the JB6 P+ cells. The results indicated that TAX is capable of opposing tumor promoter-induced carcinogenesis in JB6 P+ cells. The JB-shNrf2 cell was also established as a model for investigating the status of Nrf2 in process of the TPA-induced cell transformation. We found that no significant difference was observed between the groups induced by TAP alone and those with TAX treatment. Our results suggested that the *Nrf2* gene may play a vital role in regulating the suppressive effects of TAX on the TPA-induced JB6 P+ cell transformation.

As a pivotal activator, Nrf2 can considerably increase the transcriptional activation of detoxification and antioxidant genes by interacting with ARE [37,38]. The dietary natural compounds possessing chemopreventive potential could enhance the activities of detoxification and antioxidant enzymes via the Nrf2 signaling pathway [25]. The HepG2-C8 cell line, as an available cell line, is always used to evaluate the possible antioxidant effect of some drugs. Therefore, we determined the effects of TAX on ARE-luciferase-activity to confirm whether TAX acts as an Nrf2 inducer in HepG2-C8 cells. We found that TAX significantly increased the Nrf2-ARE activity, which suggests that the Nrf2-ARE pathway is involved in anti-cancer activity of TAX. Interestingly, Nrf2-deficient mice are susceptible to carcinogen-induced tumorigenesis [39,40]. Moreover, Nrf2 can rapidly response to oxidants by stimulating the transcriptional activation of detoxification genes, including HO-1 and NQO1. In this study, data show that TAX upregulates the mRNA and protein levels of *Nrf2* and the *Nrf2* target genes, HO-1 and NQO1 in JB6 P+ cells. It demonstrated that TAX is able to induce the nuclear translocation of Nrf2 and is likely to prevent skin cancer by inducing the expression of HO-1 and NQO1.

Epigenetic changes, such as DNA promoter methylation and histone modification, have been found to play an important role in tumorigenesis [41]. DNA methylation as a primary form of epigenetic regulation, plays a key role in maintaining DNA integrity and stability in eukaryotic animals. This modification usually occurs at the CpG islands of the promoter regions in genes. The hypermethylation of CpG islands in the promoter often results in gene silencing and could be decisive in carcinogenesis. Some specific CpG site methylations in the promoter of *Nrf2* gene have been shown to substantially decrease Nrf2 expression. Radix *Angelicae sinensis* extract and Z-ligustlide significantly reduced the level of methylation of the first five CpGs that lead to an increase in Nrf2 expression [42]. Some compounds might alter DNA demethylation and epigenetically enhance Nrf2 expression in JB6 P+ cells, including apigenin [43] and reserpine [44]. These reported indicated that some chempreventive compounds could activate the silenced *Nrf2* gene via DNA demethylation. In our study, hypermethylation of the first 15 CpG sites in the Nrf2 promoter was identified in JB6 P+ cells. The result shows that TAX decreases the methylated CpGs ratio in the promoter region of *Nrf2* gene. It was confirmed that TAX has capacity for demethylation at the first 15 CpG sites in the Nrf2 promoter region.

CpG island hypermethylation in gene promoters can be reversed by DNMT or HDAC inhibitors. In several studies, TPA-induced skin tumorigenesis was against by upregulation of Nrf2 and down-regulation of DNMTs or HDACs after treatment with curcumin or tanshinone IIA [45]. Therefore, to elucidate the specific mechanisms of TAX inhibition on the CpG sites in the Nrf2 promoter, we evaluated the protein expression of some epigenetic modification enzymes, including DNMTs and HDACs in JB6 P+ cells. In this study, the overexpression of DNMTs was observed in control cells because of the highly expression of these epigenetic modification enzymes. TAX (10 to 40 μM) was found to activate the Nrf2 signaling pathway by epigenetic regulation including the inhibition of DNMTs (subtypes of DNMT1, 3a and 3b). These results verified that TAX may decrease the methylated ratio of CpG islands in the Nrf2 promoter region by suppressing of DNMT activity. Deacetylation of histone is often correlated with “closed” chromatin and gene repression. Histone deacetylation is catalyzed by HDACs, which may play an important role in DNA repair mechanisms. Furthermore, HDACs were shown to interact with various chromatin remolding factors and transcription factors involved in transcriptional repression in occurrence of tumors. Some HDAC inhibitor has been reported to inhibit HDACs, resulting in global histone acetylation or localized hyperacetylation of histone on one specific promoter. Although studies of histone modifications are scarce in skin cancer prevention, it has been reported that elevated expression of HDACs is associated with transcriptional repression after environmental insult. In the present study, our results showed that the proteins levels of HDAC1, HDAC3 and HDAC8 were significantly decreased by TAX in JB6 P+ cells in a dose-dependent manner. The result preliminarily suggested that TAX maybe also regulates transcriptional activity of the *Nrf2* gene through histone acetylation. Thus, restoring Nrf2 expression in JB6 P+ cells, further studies are needed to elucidate the more detailed epigenetic mechanism, such as chromatin immunoprecipitation assay.

## 4. Materials and Methods

### 4.1. Chemicals and Biochemicals

TAX (purity ≥ 98%), 5-azadeoxycytidine (5-aza, purity ≥ 97%), trichostatin A (TSA, purity ≥ 98%), bacteriological agar, Eagle’s basal medium (BME) and TPA were obtained from Sigma-Aldrich (St. Louis, MO, USA). Fetal bovine serum (FBS), Dulbecco’s modification of eagle’s medium (DMEM), minimum essential medium (MEM) and trypsin-EDTA solution were obtained from Gibco Laboratories (Gaithersburg, MD, USA). The primary antibodies anti-Nrf2, anti-HO-1, anti-NQO-1 and anti-β-actin were acquired from Santa Cruz Biotechnology (Santa Cruz, CA, USA). The primary antibodies anti-DNMT (DNMT1, DNMT3a and DNMT3b) were obtained from IMGENEX (San Diego, CA, USA). The primary antibodies anti-HDAC (HDAC1, HDAC2, HDAC3, HDAC4, HDAC5, HDAC6, HDAC7 and HDAC8) were obtained from Cell Signaling Technology (Danvers, MA, USA).

### 4.2. Cell Culture and Treatment

Human hepatoma HepG2 cells were provided by Ah-Ng Kong’s laboratory (Piscataway, NJ, USA). The cells were cultured and maintained in DMEM containing 10% FBS, 100 units/mL penicillin, and 100 μg/mL streptomycin. The HepG2-C8 cell line was established by stable transfection with a pARE-T1-luciferase construct, and cultured using the same method above. JB6 P+ cells from American Type Culture Collection ATCC were maintained in MEM containing 5% FBS and 2 μg/mL puromycin. A stable Nrf2-knockdown JB6 P+ cell line (JB6-shNrf2) was established using a short hairpin RNA (shRNA) and then maintained in MEM supplemented with 5% FBS and the same concentration of antibiotics. The cells were incubated at 37 °C in a humidified 5% CO_2_ atmosphere. DMSO was used as a vehicle in all experiments at a concentration of 0.1%.

### 4.3. Cell Viability Tests

JB6 P+ cells were seeded in 96-well plates containing MEM at a density of 1 × 10^4^ cells/mL (100 μL/well), and HepG2-C8 cells were seeded in plates containing DMEM. After incubation for 24 h, the cells were treated with either DMSO or various concentrations of TAX. For JB6 P+ cells, the medium was changed every two days for the three-day and five-day treatments. Cell viability was assessed using an Aqueous One Solution Cell Proliferation (MTS) assay kit (Promega, Madison, WI, USA) according to the manufacturer’s instructions. The absorbance of the formazan product was read at 490 nm, and the cell viability was calculated and compared with the DMSO control group.

### 4.4. Luciferase Reporter Activity Assay

The stably transfected HepG2-C8 cells expressing the ARE-luciferase vector were used to study the effects of TAX on Nrf2-ARE pathways. Briefly, HepG2-ARE-C8 cells were inoculated in 12-well cell culture plates at a density of 1.0 × 10^5^ cells/well. After 24 h, the cells were treated with different concentrations of TAX (5 to 40 μM). The ARE-luciferase activity in the HepG2-C8 cells was measured using a luciferase assay kit according to the manufacturer’s protocol (Promega). Afterwards, the reporter lysis buffer was used to lyse the cells, and 10 μL of the cell lysate supernatant was analyzed luciferase activity using a Sirius luminometer (Berthold Detection System GmbH, Pforzheim, Germany). We used a bicinchoninic acid (BCA) protein assay (Pierce Biotech., Waltham, MA, USA) to determine protein concentrations. Normalization of the luciferase activity was performed based on protein concentrations.

### 4.5. Anchorage-Independent Cell Neoplastic Transformation Assay

Both the JP6 P+ and JB6-shNrf2 cells were used in the following experiments. BME containing 0.5% agar with 10% FBS was added to the bottom of six-well cell culture plates and maintained at room temperature. Subsequently, the cells were transferred to 1 mL of BME containing 0.33% soft agar layered on the top of the agar. The cells were maintained with TPA (20 ng/mL) alone or in a combination with TAX in a 5% CO_2_ incubator at 37 °C for 14 days. The cell colonies that formed in soft agar were photographed using a computerized microscope system with the Nikon ACT-1 program (Version 2.20, LEAD Technologies, Charlotte, NC, USA) and counted using the ImageJ program (Version 1.40 g, National Institutes of Health, Bethesda, MD, USA).

### 4.6. Protein Lysate Preparation and Western Blotting

After incubation for 24 h, JB6 P+ cells (5 × 10^3^ cells per 6 cm dish) were treated with various concentrations of TAX. Protein was extracted using RIPA buffer (Cell Signaling Technology), and the protein concentration was determined using the BCA kit (Pierce Biotech.). The total proteins were separated by 4% to 15% sodium dodecyl sulfate-polyacrylamide gel electrophoresis (SDS-PAGE) (Bio-Rad Laboratories, Hercules, CA, USA). After electrophoresis, the proteins transferred to a polyvinylidene difluoride (PVDF) membrane (Millipore, Billerica, MA, USA). The PVDF membranes were then blocked with 5% bovine serum albumin (BSA) in Tris-buffered saline containing 0.1% Tween 20 buffer and then blotted with primary antibodies. After washing, membranes were incubated with a horseradish peroxidase (HRP)-conjugated secondary antibodies. The blots were visualized with the Super Signal enhanced chemiluminescence (ECL) detection system was used to detect the antibody-bound proteins on the membrane. The intensity of the bands was analyzed using densitometry and the ImageJ program (Version 1.40 g, National Institutes of Health).

### 4.7. RNA Extraction and qRT-PCR

JB6 P+ cells were seeded in 6-cm diameter dishes at a density of 1 × 10^4^ cells/dish. After incubation for 24 h, the cells were treated with TAX at different concentrations for five days. Total RNA was extracted using the RNeasy Mini Kit (Qiagen, Venlo, The Netherlands). The SuperScript III First-Strand cDNA Synthesis System (Invitrogen, Waltham, MA, USA) was used to synthesize first-strand cDNA. The mRNA expression of Nrf2, HO-1 and NQO1 was determined using quantitative reverse transcription-PCR (qRT-PCR). The sequences of the primers for Nrf2, HO-1 and NQO1 were used as shown in Table 1.

### 4.8. DNA Isolation and Bisulfite Genomic Sequencing

After incubation for 24 h, the JB6 P+ cells were treated with TAX at various concentrations or 5-aza (250 nM) in MEM containing 1% FBS for 5 days. The medium was refreshed every 48 h. For combination 5-aza and TSA treatment, TSA (50 nM) was added to the medium on day 4. The cells were harvested on day 5. Genomic DNA was extracted and isolated from the treated cells using the DNA Mini Kit (Qiagen). The bisulfite conversion of genomic DNA was performed with the EZ DNA Methylation Gold Kit (Zymo Research Corp., Irvine, CA, USA) according to the manufacturer’s protocol. The bisulfite-converted DNA region was amplified by PCR using Platinum Taq DNA polymerase (Invitrogen). The DNA fragment containing the first 15 CpGs (between −863 and −1226 with the translation start site defined as position +1) in the murine *Nrf2* gene. The primer sequences 5′-AGT TAT GAA GTA GTA GTA AAA A-3′ (sense) and 5′-AAT ATA ATC TCA TAA AAC CCC AC-3′ (anti-sense) were used in this study. The PCR products were further cloned into a PCR4 TOPO vector using the TOPO™ TA Cloning Kit (Invitrogen) following the manufacturer’s instructions. The plasmids from at least ten colonies of each treatment group were randomly selected and were extracted using a QIAprep Spin Miniprep Kit (Qiagen). The target region was analyzed by sequencing (GeneWiz, South Plainfield, NJ, USA).

### 4.9. Statistical Analysis

The data are represented as the mean ± standard deviation (SD) of three independent experiments with similar results. The statistical analyses were performed using ANOVA followed by post-hoc test (Dunnett’s *t* test). The means were considered significantly different at *p* < 0.05 and *p* < 0.01.

## 5. Conclusions

This study demonstrated that TAX might alter DNA demethylation and epigenetically enhance Nrf2 expression, contributing to the prevention of the neoplastic growth of JB6 P+ cells. The finding suggests that the preventive potential of TAX against skin carcinogenesis is mediated through a novel molecular mechanism. TAX may play an important role in preventing skin cancer by epigenetic modifications. However, additional studies on animal models of skin cancer should be further investigated. The nanodispersion of TAX can be used via oral administration, which could improve the bioavailability of taxifolin significantly [46].

## Figures and Tables

**Table 1:** The primers of Nrf2, HO-1, NQO1 and β-actin for quantitative reverse transcription-PCR (qRT-PCR).

Gene	Direction	Oligonucleotide Sequence 5′–3′
*Nrf2*	sense	5′-AGCAGGACTGGAGAAGTT-3′
	antisense	5′-TTCTTTTTCCAGCGAGGAGA-3′
*HO-1*	sense	5′-CCTCACTGGCAGGAAATCATC-3′
	antisense	5′-CCTCGTGGAGACGCTTTACATA-3′
*NQO1*	sense	5′-AGCCCAGATATTGTGGCCG-3′
	antisense	5′-CCTTTCAGAATGGCTGGCAC-3′
*β-actin*	sense	5′-CGTTCAATACCCCAGCCATG-3′
	antisense	5′-GACCCCGTCACCAGAGTCC-3′

## Supplementary Materials

Supplementary materials can be found at www.mdpi.com/1422-0067/18/7/1546/s1.

## Abbreviations

ARE	Antioxidant response element
ATCC	American type culture collection
DMEM	Dulbecco’s modification of Eagle’s medium
DMSO	Dimethyl sulfoxide
DNMT	DNA methyltransferase
BME	Eagle’s basal medium
FBS	Fetal bovine serun
HDAC	Histone deacetylase
Keap1	Kelch-like ECH-associating protein 1
HO-1	Heme oxygenase-1
MEM	Minimum essential medium
MTS	3-(4,5-dimethylthiazol-2-yl)-5-(3-carboxymethoxyphenyl)-2-(4-sulfophenyl)-2H-tetrazolium
NQO1	NAD(P)H quinone oxidoreductase 1
Nrf2	Nuclear factor erythroid-2 related factor 2
ROS	Reactive oxygen species
SD	Standard deviation
TAX	Taxifolin
TPA	12-*O*-tetradecanoylphorbol-13-acetate
TSA	Trichostatin A
UV	Ultraviolet
