# Supplementary material for: Taxifolin Activates the Nrf2 Anti-Oxidative Stress Pathway in Mouse Skin Epidermal JB6 P+ Cells through Epigenetic Modifications
Source: Int J Mol Sci. 2017 Jul 17;18(7):1546. doi: 10.3390/ijms18071546 (PMC5536034; doi:10.3390/ijms18071546)
Supplement: Supplementary file 1 [file ijms-18-01546-s001.pdf]

Supplementary File:

A: Original
ATACTTACATTACAATTTATAGCAACAAAATTAGTTATGAAGTAGCAGCAAAA
ATACTTTTCTAGTTGGAGGTCACCACAACACGAACTATATTAAAGGGTCGCTG
CATTACGACGGTTGAGAAACGCTGCTCTAAAAACAGCTTGAGAGGTGGGGCC
TTATGAGATCATACCTCAAAATTCCCAAATAGAGTAAGTACGTGTAAAGGAAC
CCTGAGAGTCTTAGAATCCACTTCGGTGATTCTGTTAGAGCTGTGCCCGCTTGC
TGGGCTCTGGGTGGATGAAGTGATACTGAGTGACCGCTTGGGCCCCGAGCCC
AGGGCACGTGGGAGAAGTGGAGGGCCTTCGAGTAAGCGAGCACGAGTTTGC
AGCGTGGACTCATCCATCTCCCTGGGGC

B: CT-converted and methylated CpG (100% methylation template)
ATATTTATATTATAATTTATAGTAATAAAATTAGTTATGAAGTAGTAGTAAAAAT
ATTTTTTTAGTTGGAGGTTATTATAATACGAATTATATTAAAGGGTCGTTGTATTA
CGACGGTTGAGAAACGTTGTTTTAAAAATAGTTTGAGAGGTGGGGTTTTATGA
GATTATATTTTAAAATTTTTAAATAGAGTAAGTACGTGTAAAGGAATTTTGAGA
GTTTTAGAATTTATTTCGGTGATTTTGTTAGAGTTGTGTTCGTTTGTTGGGTTTTG
GGTGGATGAAGTGATATTGAGTGATCGTTTGGGTTTCGAGTTTAGGGTACGTG
GGAGAAGTGGAGGGTTTTCGAGTAAGCGAGTACGAGTTTGTAGCGTGGATTT
ATTTATTTTTTTGGGGT

C: CT-converted and unmethylated CpG (100% unmethylation template)
ATATTTATATTATAATTTATAGTAATAAAATTAGTTATGAAGTAGTAGTAAAAAT
ATTTTTTTAGTTGGAGGTTATTATAATATGAATTATATTAAAGGGTTGTTGTATTA
TGATGGTTGAGAAATGTTGTTTTAAAAATAGTTTGAGAGGTGGGGTTTTATGA
GATTATATTTTAAAATTTTTAAATAGAGTAAGTATGTGTAAAGGAATTTTGAGA
GTTTTAGAATTTATTTTGGTGATTTTGTTAGAGTTGTGTTTGTTTGTTGGGTTTTG
GGTGGATGAAGTGATATTGAGTGATTGTTTGGGTTTTGAGTTTAGGGTATGTGG
GAGAAGTGGAGGGTTTTTGAGTAAGTGAGTATGAGTTTGTAGTGTGGATTTAT
TTATTTTTTTGGGGT

Partial base sequence in the Nrf2 promoter region was shown as above, which contain the first 15 CpG sites. The first 15 CpG sites are highlighted in red in the above sequence. A: Original partial sequence of Nrf2 promoter region under normal physiological conditions; B: the base sequence is a 100% methylation template of the first 15 CpG sites in Nrf2 promoter region by bisulfite genomic sequencing; C: the base sequence is a 100% unmethylation template of the first 15 CpG sites in Nrf2 promoter region by bisulfite genomic sequencing.
